# Non-high altitude methods for rapid screening of susceptibility to acute mountain sickness

**DOI:** 10.1186/1471-2458-13-902

**Published:** 2013-09-30

**Authors:** Han Song, Tao Ke, Wen-Jing Luo, Jing-Yuan Chen

**Affiliations:** 1 Department of Occupational and Environmental Health, School of Preventive Medicine, Fourth Military Medical University, No.169, Changlexi Road, Xi’an, Shaanxi 710032, China

**Keywords:** Acute mountain sickness, Susceptibility, Screening techniques

## Abstract

**Background:**

Acute mountain sickness (AMS) refers to the cerebral abnormalities typically triggered by exposure to hypobaric hypoxia at high altitude. Although AMS is not often life threatening, it can seriously impact health quality and decrease productivity. Thus, detection of potential susceptibility to AMS has become important for people arriving at high-altitude plateaus for the first time, including laborers and military staff. The aim of this review was to examine techniques which efficiently assess the susceptibility to AMS prior to exposure to high altitude.

**Methods:**

By searching online databases, we retrieved studies with associations between AMS and methods to detect the susceptible people who were not exposed to high altitudes. Studies reporting significant correlation coefficients between screening methods and AMS scores were included.

**Results:**

Several screening techniques of AMS susceptibility were found including cold pressor test, heart rate variability, and lung functions. Of these markers, heart rate variability was positively associated with AMS scores, while the rest were negatively associated with AMS scores.

**Conclusions:**

We identified three physiological markers that were significantly associated with the risk of AMS. Although it is well known that simple sea level tests are not really helpful in predicting AMS currently, these markers, to some degree, may be employed as references in predicting susceptibility.

## Background

Acute mountain sickness (AMS) describes the cerebral syndromes that can develop in unacclimatized people shortly after ascent to high altitude above 2500 m [1]. The reported prevalence of AMS varies widely because of different ascent profiles. For example, in Summit County of Colorado, the incidence of AMS was 22% at altitudes of 1850–2750 m [2] and 42% when ascending to 3000 m [3], and was as high as 75% of people attempting ascent of Mount Kilimanjaro (5984 m) [4]. In laborers [5] and soldiers [6], the incidence of AMS was much higher due to the requirements for laboring or completion of missions under hypobaric hypoxia. Under some conditions, AMS can result in oliguria, retinal hemorrhage, ataxia, and coma [7], which can ultimately proceed to life-threatening high altitude pulmonary edema (HAPE) and high altitude cerebral edema [1]. With the increasing demand for economic development, a large number of people will be at risk of high altitude illness when they first reach high altitude regions [8]. Thus, it is critical to screen people with potential susceptibility to AMS, particularly soldiers and workers, as they will be at higher risk due to exercise in hypoxic conditions, which was shown to increase the incidence and severity of AMS compared with a similar sedentary trial [9].

Several risk factors for AMS have been identified, including speed of ascent above 300 to 500 m per day in the acclimatization period [10], previous history of AMS or HAPE [11], obesity [12], migraine [13], persistence of a patent foramen ovale [14], residence at an altitude below 900 m [2], exertion [9], certain preexisting cardiopulmonary conditions [15], and various biochemical and genetic factors [16]. However, the incidence and severity of AMS not only depend on the above factors, but also on the individual’s physiological susceptibility which may as well associate with AMS [11]. Some physiological parameters including arterial oxygen saturation [17] and mean pulse rate [18] that may influence AMS rely on pre-exposure to a high altitude or simulated conditions. Nevertheless, it is inconvenient and unrealistic for people to experience exposure to hypobaric hypoxia prior to going to high altitude for the first time. Hence, our overall objective is to review the studies that explored methods for prediction of AMS susceptibility to develop rapid screening techniques for susceptible subjects, though test results may have different meaning for various sub-populations, not depending on pre-exposure to altitude or simulated-altitude.

## Methods

### Searching strategy

In accordance with the preferred reporting items for systematic reviews (PRISMA) statement [19], a computerized literature search was performed within Medline (PubMed), ISI web of science, EMBASE, Chinese Biomedical Disk, and the Chinese Journal Full-test Database (up to April 2012). Both English and non-English language articles were examined. The full text research articles that could not be downloaded from the databases were obtained via e-mail with the authors or by hand searching.

The retrieval key words used were: Altitude Sickness, hypoxia, susceptibility, and screening techniques.

### Inclusion and exclusion criteria

Inclusion criteria:

1. Studies should report the detection of susceptibility to AMS not depending on exposure of high altitude or simulated.

2. Screening techniques should be convenient and have low cost such as physiological parameters, which could be used by large populations.

3. The sample size must be larger than 30 cases.

4. Results of the studies must include data representing the association between score of AMS and predicted methods, such as correlation efficient and *P*-value.

Exclusion criteria:

1. Animal experiment and unrelated studies, such as pathological and physiological studies on the association between AMS and its risk factor.

2. Screening methods rely on complicated and inconvenient techniques such as detection of gene polymorphism.

3. Study publications don’t contain the minimum information necessary to obtain correlation efficient and corresponding 95% confidence intervals.

4. Studies focused on detection of susceptibility to AMS at high altitude or simulated.

### Definition of AMS

The Lake Louise scoring system, a well-validated standard [20], was used for the diagnosis of AMS. Subjects with the presence of headaches and at least 1 of the following symptoms were determined to have AMS: gastrointestinal upset (loss of appetite, nausea, and vomiting), fatigue/weakness, dizziness/light-headedness, or insomnia (more than just the usual frequent waking).

### Quality assessment of included studies

Two reviewers assessed quality of studies independently using a tool based on the criteria described by Jadad for prospective studies. Assessment discrepancies between the reviewers were resolved through discussion and double-check.

As for prospective study, the following items were evaluated: (1) randomization procedures, (2) matching procedures, (3) blinding procedures, (4) analysis of dropouts, (5) reliability and validity of assessment instruments, (6) control for cointerventions, (7) comparability of baseline patient characteristics, (8) control for amount of therapy, and (9) applied statistics etc.

## Results

**Figure 1:**
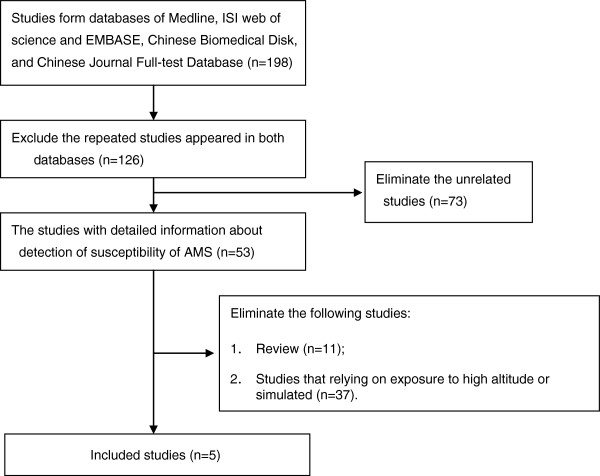
The flow chart.

From 198 retrieval studies, 5 studies from China and Nepal were finally included (Figure 1). Data in all included studies were obtained through following up and during process of research both researchers and subjects were exposed to hypobaric hypoxia.

### General characteristics

Table 1 presents the characteristics of included studies. All studies had a prospective longitudinal design: one was from Nepal, the rest were from China, where abundant territory belongs to high altitude mountain or plateau and only one used the hypobaric chambers to stimulate the true environment. Altitude of departure were all less largely than destination. Sample size, though large than 30, was less than other clinical studies and volunteers and soldiers, in whom males took advantages, were main subjects. However, the intervals of ages of subjects reported by Huang LL et al. [21] were 30 years old larger than the others. Among the five studies focused on detection of susceptibility to AMS, two on HRV, two on CPT and two on lung functions. Furthermore, Lake Louise scoring system, a well-validated standard, was used for the diagnosis by Huang LL et al. [21] and Diagnostic Criteria of High Altitude Disease in China, which was brought into effect in China during the 1990s and introduced to the world through translation by John B. West in 2010, was employed by the remaining studies.

**Table 1:** Characteristics of included studies

**Study**	**Research place**	**Altitude of departure**	**Altitude of destination**	**Sample size**	**Subjects**	**Sexes**	**Ages**	**Screening method**	**Measures of AMS**	**Statistical index**	***P *value**
Huang HH [21]	Nepal	<500 m	3440 m	32	Trekkers	Males & Females	49.3 ± 2.3	HRV	L	Odds Ration	<0.05
Tian KX [35]	Tibet	500 m	3700 m	99	Volunteers	Males	18.2 ± 0.8	HRV & CPT	C	Correlation coefficient	<0.01
Long M [29]	Hypobaric chamber	242 m	4500 m	43	Soldiers	Males	19.02 ± 0.96	CPT	C	*t* value	<0.05
Zhou QQ [25]	Tibet	300	3685 m	113	Soldiers	Males	19 ± 1	FEV1	C	Correlation coefficient	<0.01
Wang L [46]	Tibet	1500	4900 m	60	Soldiers	Males	19.8	FVC/BSA	C	*t* value	<0.01

*Abbreviations*: *L* Lake Louise scoring system, a well-validated standard, *C* Diagnostic Criteria of High Altitude Disease in China, which was brought into effect in China during the 1990s and introduced to the world through translation by John B. West in 2010.

### Quality assessment

The quality scores of the included studies are summarized in Table 2. Because of research difficulties and considerations of ethics, the included population studies about effects of high altitude exposure on human bodies were basically belonged to quasi-experiment compared with randomized controlled trials. Therefore, evidence degree was lower than randomized controlled trials but higher than observational studies. Further, due to lack of experimental controls, the five studies totally employed own controls before and after exposure to hypoxia.

**Table 2:** Quality assessment of included studies

**Trials**	** Criteria**																				**Total score**			
	**A**	**B**	**C**	**D**	**E**	**F**	**G**	**H**	**I**	**J**	**K**	**L**	**M**	**N**	**O**	**P**	**Q**	**R**	**S**	**T**	**U**	**V**	**W**	**X**
Huang HH [21]	0	0	1	0	1	0	1	1	0	1	1	1	1	0	0	0	1	1	1	0	1	12	9	1.33
Tian KX [35]	0	0	1	0	1	0	1	1	0	1	1	1	1	0	0	0	1	1	1	1	1	13	8	1.63
Long M [29]	0	0	1	0	1	0	1	1	0	1	1	1	1	0	0	0	0	1	1	0	1	11	10	1.1
Zhou QQ [25]	0	0	1	0	1	0	1	1	0	1	1	1	1	0	0	0	1	1	1	1	1	12	9	1.33
Wang L [46]	0	0	1	0	1	0	1	1	0	1	1	1	1	0	0	0	0	1	1	0	1	11	10	1.1

*Abbreviations*: *A* Randomized controlled trial, *B* Method of randomization appropriate, *C* Study prospective, *D* Double-blind, *E* Inclusion and exclusion criteria, *F* Method of double-blinding appropriate, *G* Participants selection described, *H* Comparison of treatment and control or before experiment and after, *I* Description of withdrawals, *J* Models adequate, *K* Objectives defined, *L* Outcome defined, *M* Sample size justified, *N* Description of the co-interventions, *O* Other care identical, *P* Source of bias addressed, *Q* Raw data available, *R* Statistical methods described, *S* Published (not just abstract), *T* Financial support described, *U* quasi-experiment, *V* Number of yes, *W* Number of no, *X* Ratio yes/no, *1* yes, *0* no/not mentioned, *N* not applicable.

### Test procedure

The cold pressor test (CPT) is a standardized procedure initially used to induce a cardiovascular response in humans [22]. Subjects are asked to immerse one hand to just above the wrist for 1 min in ice water (4-5°C; 39-41°F). During this period, blood pressure is recorded on the opposite arm at 15 s intervals, and the highest reading designated the peak or ceiling blood pressure. The difference between peak and basal blood pressures determines the level of vascular reactivity [23].

Heart rate variability (HRV) is assessed in the participants resting in supine position on mats on the floor, separated from the rest of the clinic by a divider. The participants wear a chest strap from a heart-rate monitor watch. After a 5-min rest period, heart rate is collected on a beat-by-beat basis for 10 min. HRV consists of a standard time domain including the standard deviation of R-R intervals, the root mean square of the mean differences in successive R-R intervals, and the percentage of successive R-R intervals that differs by more than 50 ms. Frequency domain analysis is performed by using nonparametric fast Fourier transform and autoregressive modeling, which contains spectral powers at low frequency (LF), high frequency (HF), very low frequency and total power [24].

Pulmonary functions are tested with Sensor Medics Vmax229D Pulmonary function instrument. Standardized gas is employed to determine the standard before test. Detection index includes flow-volume loop, maximal voluntary ventilation, forced expiratory volume in 1 second (FEV1), forced vital capacity (FVC) and peak expiratory flow. Each measure is repeated twice, and the average value for statistical analysis [25].

## Discussion

### Autonomic nervous system

A marked increase in peripheral sympathetic activity is a common feature of mountain sickness [26]. Increased sympathetic activity is part of the integrated physiological response to a hypoxic stimulus [27]. Interestingly, subjects with AMS had an abnormal pattern of cardiovascular variability compared with subjects without AMS [28]. Thus, assessment of the degree of sympathetic activity of the automatic nervous system may be used to evaluate susceptibility to AMS [29].

### Cold pressor test

The cold pressor test (CPT) involves placing a hand or forearm in cold water, a stimulus that produces a slowly mounting pain of mild to moderate intensity, and being terminated by voluntary withdrawal of the limb. The CPT has been used in many studies of pain, autonomic reactivity, and hormonal stress responses [30]. This acute stress test used to evaluate the initial hemodynamic response to a stimulus also represents an index of integrity of sympathetic functions at the efferent level. For example, CPT was reported to induce a large sympathetic discharge in the spinal cord and terminal endings of the sympathetic nervous system [31]. Thus, CPT is widely used to evaluate the sympathetic neural influence on circulation in normal humans [32].

In healthy populations, the hemodynamic changes observed in response to CPT were similar when measured at sea level and after ascending to high altitude [33]. Viswanathan originally reported that blood pressure changes could be used to predict AMS with CPT [34]. Meanwhile a more recent study reported that subjects unsusceptible to AMS exhibited a higher blood pressure and heart rate response to CPT (r = −0.35, P = 0.01). This study found that CPT in 99 healthy young males was examined at plain and their AMS scores were investigated in successive 5 days after exposure. The increase in heart rate was less in moderate to severe AMS group [(7.57 ± 8.22)b/min] than in very little response group [(25.47 ± 19.26)b/min)] after CPT (P < 0.05) [35]. It seemed that autonomic nervous system (ANS) reflected by CPT response played an important role in the progress of the AMS. Function of ANS may be inhibited after exposure to high altitude and subjects with weaker ability of ANS regulation were prone to be susceptible to AMS. Test of ANS with CPT may be helpful in distinguishing susceptible ones at lower altitudes. Also, another study found that there existed significant differences of CPT before entering stimulated high altitude between AMS and non-AMS [29]. Thus, further studies on the use of CPT for evaluation the risk of AMS are required.

### Heart rate variability

The autonomic nervous system plays a role in the modulation of the oscillatory behavior of the cardiovascular system [36]. Power spectral analysis of HRV, a new noninvasive measurement to evaluate the sympathovagal balance between the sympathetic and parasympathetic influence on the heart [37], is a recognized tool to quantify these oscillatory components. Specific spectral components of HRV are considered to be associated with autonomic modulation of the heart [38]. In addition, increased sympathetic activity during acute exposure to hypobaric hypoxia at high altitude is a common presentation of AMS. Thus, some studies, as depicted below, have explored whether the sequential changes of specific spectral components of HRV could be used as an early prediction for the presentation of AMS after trekking from sea level via low to high altitude.

The time domain and frequency domain in HRV could change significantly after simulated or actual high altitude exposure [28,39]. Further, significant differences were found in total variance of R-R interval at high altitude between subjects with and without AMS [28]. HRV also plays an important role in predicting AMS at low altitude. For example, Huang et al. reported that subjects with both HF% <20% and a LF: HF ratio >1.3 measured at 1317 m had an odds ratio of 7.00 (95% confidence interval, 1.11-44.06; *P* = 0.047) for developing AMS at 3440 m. A total of 32 subjects (10 men and 22 women) were recruited and a 12-day itinerary by trekking to the Namche Bazaar in Nepal in this study. It may implicate that predominantly sympathetic modulation on the heart in the exposure of mild hypoxia may be prone to suffering AMS in more severe hypoxic condition. The suggested reasonable low altitude measurement of HRV that would be useful in predicting the risk of AMS at high altitudes would be around the height of 1300 m to 1400 m [21].

Similar results were reported by Tian et al. [35], where trend of LF/HF and normalized LF decreased associated with lower AMS scores and normalized HF increased along with higher AMS scores, found that AMS was positively correlated with LF:HF (*r* = 0.437, *P* < 0.01). The LF/HF ratio has gained wide acceptance as a tool to assess cardiovascular autonomic regulation where increases in LF/HF are assumed to reflect a shift to “sympathetic dominance” and decreases in this index correspond to a “parasympathetic dominance [40]”. Thus, the positive relationship may be explained by that people with dominant sympathetic activity at plain would be more susceptible to AMS. Overall, these data suggest that HRV might be useful for screening of people at risk for AMS.

### Pulmonary functions

A drop in the oxygen pressure of the arterial blood leads to hypoxic stimulation of peripheral chemoreceptors primarily in the carotid and aortic bodies, causing an increase in alveolar oxygen pressure and arterial oxygen pressure. This effect is known as the hypoxic ventilatory response [41], and the involuntary increase in the ventilatory response is by far the most important feature of acclimatization [42]. Bartsch et al. also reported that alveolar–arterial oxygen pressure difference was higher, and arterial oxygen pressure was lower, in AMS subjects compared to controls, suggesting pulmonary dysfunction in AMS [43]. Further, two previous studies found decreased vital capacity with preserved FEV1/FVC ratios in subjects with AMS [44,45]. Nevertheless, few studies have utilized the pulmonary functional parameters such as FEV1 and FVC in predicting AMS at sea level. As an example, Zhou et al. showed that FEV1 was negatively correlated with the AMS score (*r* = −0.244, *P* = 0.009). In this study, 113 males were recruited from their native places in plain. Their lung function was determined at the altitude of 300 m before air transportation. Scores of AMS were recorded by medical staffs 2 or 3 days after their arrival at the altitude of 3 658 m and correlative analysis of each lung functional index and scores of AMS was subsequently conducted [25]. Another study found that FVC/body surface area (BSA) had the predicted role when FVC/BSA > 3 liter per square meter [46]. Many studies have also used limited samples sizes because of the difficulty in transporting people to high altitude. Nevertheless, overall these data suggest that pulmonary functions, which partly decide hyperventilatory capacity, may be used to predict altitude sickness [47].

### Other methods and limitations

Other methods for predicting AMS not included in this study have been reported. For example, Missoum et al. found that anxiety prior to ascent could predict AMS symptoms psychologically [48]. However, the questionnaires and scales used for measuring anxiety in that study were not sensitive to the symptoms of clinical anxiety and panic [49]. Other studies have examined indicators of AMS during ascent exposed to hypobaric conditions such as arterial oxygen saturation [50]. Although these parameters are more accurate as they were tested in actual or simulated situations, the costs of such studies are high. Nevertheless, such indexes may be used to monitor for AMS during transport to altitude to allow prompt treatment in susceptible people [51].

There are several limitations of our study. Our indicative system may not reflect the full ability of adaption to hypoxemia as other indicators may have been overlooked. Further, when constructing a predictive model, there may exist colinearity between parameters such as CPT and HRV, which are both autonomic nervous system indicators. This potential bias may reduce the accuracy of predictions for AMS.

## Conclusions

In this review, we found three significant markers including, CPT, HRV and lung functions, to some degree, may be employed as references in predicting susceptibility, though none sea level tests do not really come into service in predicting AMS to date without reliance on simulated or actual exposure to high altitudes (Table 3). Further, we should also highlight that these associations were weak because all correlation coefficients in the analyzed studies were under 0.5. Future studies should explore two aspects to decide whether these physiological variables were effective or not. Firstly, we should find whether these three markers of susceptible subjects could be turned into the normal level of unsusceptible ones after taking some medicine which could prevent or cure AMS. In addition, prospective cohort studies may be carried out to ensure practicability and feasibility of these variables which may also be utilized to develop a mathematical predictive model.

**Table 3:** Correlation coefficients between indicators and AMS

**Parameters**	**Correlation coefficient (*r*)**	***P *value**	**Instructions**
Cold pressor test	−0.35	0.01	Published
Heart rate variability	0.437	<0.01	Published
Lung functions	−0.244	0.009	Published

From a public health perspective, our goal is to develop simple and rapid screening tests for assessing susceptibility to AMS and to be used in people that will work or be deployed to complete missions at high altitudes. Thus, these indicators must objectively reflect the ability of resistance to hypoxia, while the methods should be convenient and have low cost [52]. Finally, these findings might provide a valuable reference to guide highland health services so that medical cost may be reduced and life quality of subjects may be improved.

## Abbreviations

AMS: Acute mountain sickness; BSA: Body surface area; CV: Chest volume; CPT: Cold pressor test; FEV1: Forced expiratory volume in 1 s; FVC: Forced vital capacity; HAPE: High altitude pulmonary edema; HF: High frequency; HRV: Heart rate variability; LF: Low frequency; ANS: Autonomic nervous system.

## Competing interests

The authors declare that they have no competing interests.

## Authors’ contributions

HS and WL designed the study. HS collected and analyzed the data and drafted the manuscript. TK collected data and provided expert statistical advice. HS researched and collected articles. JC provided human resource support and research advice. All authors were involved in critical evaluation and editing of the manuscript, and read and approved the final version.

## Pre-publication history

The pre-publication history for this paper can be accessed here:

http://www.biomedcentral.com/1471-2458/13/902/prepub
